# Environmental Monitoring for Enteroviruses in Maputo, Mozambique—2018

**DOI:** 10.3390/pathogens11050527

**Published:** 2022-04-29

**Authors:** Diocreciano Matias Bero, Sheila António Nhassengo, Ivanildo Pedro Sousa, Jr., Silas Oliveira de Sousa, Raiana Scerni Machado, Amanda Meireles Nunes Dias, Cristiane de Sousa Ferreira, Fernanda Marcicano Burlandy, Nilsa de Deus, Edson Elias da Silva

**Affiliations:** 1 Instituto Nacional de Saúde, Bairro da Vila de Marracuene, EN1, Parcela No 3943, Maputo P.O. Box 264, Mozambique; sheilanhassengo.2016@gmail.com (S.A.N.); ndeus1@yahoo.com (N.d.D.); 2 Laboratório de Enterovírus, Instituto Oswaldo Cruz, Fundação Oswaldo Cruz, Rio de Janeiro CEP 21040-210, Brazil; ivanildo.sousa@ioc.fiocruz.br (I.P.S.J.); silasdeoliveira@gmail.com (S.O.d.S.); raianascerni22@hotmail.com (R.S.M.); meirelesa-manda@hotmail.com (A.M.N.D.); cris_ilazyr@yahoo.com.br (C.d.S.F.); fburlandy@ioc.fiocruz.br (F.M.B.); edson.fiocruz@gmail.com (E.E.d.S.)

**Keywords:** environmental surveillance, enterovirus, wastewater, poliovirus

## Abstract

Due to the possibility of wild poliovirus importation from endemic regions and the high circulation of vaccine-derived poliovirus type 2 in the African region, Mozambique implemented a surveillance program to monitor the circulation of enteroviruses in the environment. From January to November 2018, a period that immediately preceded the cVDPV outbreak in Africa, 63 wastewater samples were collected from different areas in Maputo city. A total of 25 samples (39.7%) were positive based on cell culture isolation. Non-polio enteroviruses were found in 24 samples (24/25; 96%), whereas 1 Sabin-related poliovirus was isolated. Neither wild nor vaccine-derived poliovirus was detected. High circulation of EVB species was detected. Environmental surveillance in the One Health approach, if effectively applied as support to acute flaccid paralysis, can be a powerful aid to the public health system to monitor poliovirus besides non-polio enteroviruses in polio-free areas.

## 1. Introduction

Enterovirus (EVs) (family *Picornaviridae*, genus *Enterovirus*) are nonenveloped, small, icosahedral-shaped viruses [1]. Even though they have been classified into 15 species (EV A–L and human rhinoviruses A–C), only EV A–D species and rhinoviruses are known to cause human infection [1]. These viruses cause a wide spectrum of diseases in humans, including febrile exanthems, respiratory infections, acute hemorrhagic conjunctivitis, aseptic meningitis, encephalitis, acute flaccid paralysis (AFP), myocarditis, gastroenteritis, etc. [1,2]. EVs are predominantly transmitted through the fecal–oral route and are regularly discharged into the environment with human feces. Enteroviruses are distributed globally, particularly in tropical regions where poor hygiene conditions play critical roles in the efficiency of viral transmission.

Environmental surveillance (ES) has been an effective approach for monitoring the circulation of human or animal agents and assessing the extent or duration of outbreaks in several communities, providing information to tailor public health policies [3,4,5]. Enteric viruses are shed by asymptomatic individuals as well as symptomatic patients [6]. Furthermore, the vast majority of the fecal–oral-transmitted viruses are highly stable and persist in water, food, and environmental surfaces for a long time [7,8,9]. Monitoring of sewage or wastewater has been an important component of the surveillance system, particularly in developing countries [10].

Within this context, the World Health Organization (WHO) launched the Polio Eradication and Endgame Strategic Plan, 2013–2018, for which the ES should play a critical role in monitoring and providing evidence about polioviruses’ circulation, including vaccine-derived polioviruses (VDPVs) following the discontinuation of the oral polio vaccine (OPV) [11].

In Mozambique, the last case of paralytic poliomyelitis caused by indigenous polioviruses occurred in 1993, and vaccination coverage against polio has increased in the last few years since its introduction, although it remains below 90% in the country [12]. Since then, poliomyelitis has been controlled mostly by using the trivalent oral polio vaccine (tOPV) [12]. In April 2016, the tOPV was replaced by the bivalent oral polio vaccine (bOPV) [12]. Recently, outbreaks related to VDPV have been reported in 16 countries in several African regions, including Mozambique [11,13]. Thus, the risk of VDPV circulation remains elevated, and the ES may play a significant role in supporting the WHO global action plan for poliomyelitis eradication [10,11]. As the information about the circulation patterns of EVs in Mozambique is limited [14], mainly due to the absence of a specific EV surveillance system, we decided to carry out the present study to monitor the presence of NPEVs, VDPVs, and polioviruses in sewage samples in the city of Maputo from January to November 2018.

## 2. Results

From the 63 sewage samples collected at sites, 25 (39.7%) yielded enterovirus characteristic CPE only in the RD lines (Table 1), while 1 sample also showed CPE in L20B. Regarding the monthly distribution, we observed that the EV detection in all sentinel sites occurred only throughout March, as well as the identification of more than two EV types. The site with the highest number of isolates was ETAR (13 isolates), followed by Marginal Avenue (9 isolates) and November 10th Avenue (3 isolates) (Table 1).

Molecular genotyping from the 25 positive samples revealed that all NPEVs belonged to Enterovirus B species as follows: coxsackievirus B3 (36%; 9/25), echovirus 11 (28%; 7/25), coxsackievirus B5 (12%; 3/25), echovirus 7 (12%; 3/25), echovirus 6 (4%; 1/25), and enterovirus B75 (4%; 1/25).

The poliovirus isolated in L20B cells was characterized as Sabin-like 1 (SL PV1, Enterovirus C species) and revealed only one substitution at residue A^316^ (A → G) within the complete VP1 gene. Neither wild-type poliovirus nor VDPV was found in any environmental sample collected in three of the country’s most densely inhabited areas during the period of the study (January–November 2018).

**Figure 1:**
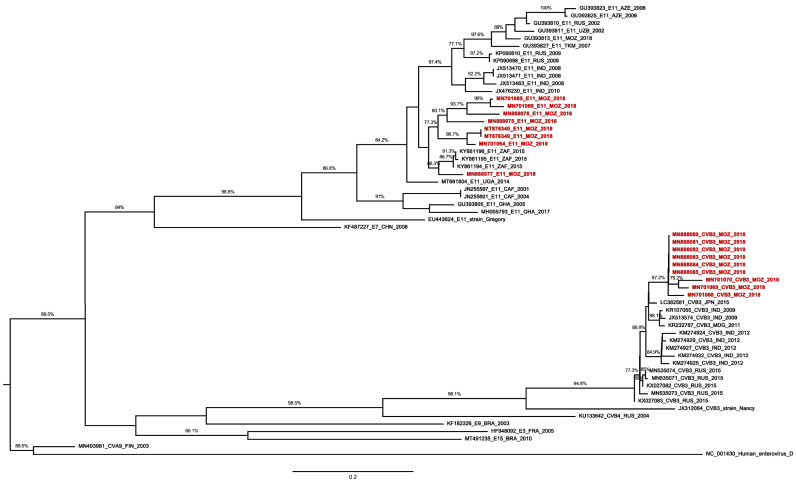
Phylogenetic analysis based on 396nt of E11 and CVB3 VP1 sequences of Mozambican isolates (colored) obtained from sewage samples and other sequences available in the GenBank database. The phylogenetic tree was reconstructed using a maximum likelihood algorithm (RAxML), with a GTR + I + G nucleotide substitution model, and edited in FigTree version 1.4.3. The strain name, year of sampling, and GenBank accession numbers are presented. The outgroup is represented by EV D species.

In order to investigate genetic variability, partial VP1 sequences of Mozambican isolates were compared with sequences available at GenBank. CVB3 isolates showed a clear genetic relationship (Figure 1) with strains isolated in India (2009) and Japan (2015). Regarding E11, phylogenetic analysis indicated the existence of different genetic clusters related to EV types identified in South Africa in 2015 (Figure 1).

## 3. Discussion

As a supplement to AFP surveillance, the WHO has incorporated environmental poliovirus surveillance in the Global Polio Eradication Initiative’s Strategic Plan [2]. Indeed, ES is a sensitive approach for detecting silent circulation of the wild-type and vaccine-derived polioviruses, which has helped to understand the circulation patterns of NPEVs in different countries [2,4]. During this study, a total of 63 samples were screened, yielding 25 (39.7%) EV-positive isolates. The positivity rate reported in this study is within the expectations of the WHO-recommended enterovirus surveillance guidelines, which suggest that the acceptable detection rate should be between 5% and 25% [2]. Similar results were obtained in previous studies conducted in Nigeria, India, and South Africa, which reported 34.6%, 40.4%, and 42.5%, respectively [15,16,17]. On the other hand, two other studies have reported higher values, Brazil and Senegal (70%) [8,18].

In the present study, EV positivity from ES samples was assessed through cell culture isolation using RD and L20B cell lines. The vast majority of the isolates belonged to the Enterovirus B species. These results are in agreement with previous studies performed in Georgia and Ghana [18,19]. Interestingly, the lack of NPEV Enterovirus C (EV C) species could be possibly justified by the absence of the use of the HEp-2c cell line in the laboratory procedure. In fact, some studies have demonstrated that the use of HEp-2c cells increased the number of EV C isolates, suggesting that the absence of this cell line in an isolation routine can lead to underestimating EV C isolation [20,21,22]. Conversely, the high detection rate of EV B species may be due to the use of RD cells for virus isolation, which are highly sensitive to EV B but less susceptible to EV A and non-polio EV C [21,22,23].

Only one PV1 was isolated and identified through ES. This Sabin-related poliovirus was probably excreted by individuals recently immunized with the oral poliovirus vaccine, which is still used in Mozambique within the immunization schedule. The single-point mutation found in this Sabin-like isolate suggests a very limited circulation in the community [24]. It is worth mentioning that our samples were taken prior to the current outbreak on the African continent **.** Although the African Regional Certification Commission certified Africa as polio-free in 2020, a recent WPV1 case in Malawi (2021) [25,26] and continuous VDPV circulation (2018 in Mozambique and 2019 in Ghana) [11,13,27] highlight the need to tailor current surveillance strategies (including ES, which has not been implemented in our country) to timely monitor the emergence/re-emergence of polioviruses.

Enteroviruses were isolated every month of sample collection, and no apparent peak was evident during this period. Seasonality patterns are less evident in tropical areas because infections occur throughout the year [28]. EV-B75 was reported at one sentinel site. This EV type has been reported as being associated with AFP cases [15,29], and it is not frequently found in environmental samples. This reinforces the importance of this study and highlights the need to keep ES active within the public health system to detect the emergence of uncommon strains with neurotropic potential.

Coxsackievirus B3 and E11 were the main detected EVs from environmental samples. These EV types, particularly CVB3, have not been reported in previous studies in many countries of Africa [14,16,21]. However, it is worth mentioning that the detection rate of each EV type depends on many factors, such as climatic factors and population density.

## 4. Materials and Methods

The city of Maputo is geographically located in the southern region of Mozambique, covers an area of 34,677 km^2^, and has an estimated population of 1,120,867 inhabitants [30]. A total of 63 wastewater samples were collected from the largest sewage system in Maputo, consisting of three collection sites—Infulene sewage treatment plant (ETAR), November 10th Avenue, and Marginal Avenue dump effluents. Samples were transported at 4 to 8 °C to the Virology Laboratory of the Instituto Nacional de Saúde of Mozambique. After collection, sewage samples were separated into two aliquots of 500 mL each. One aliquot was stored at −20 °C, whereas the other was used for concentration to 5 mL volumes using the silica adsorption method [8,31].

Concentrated samples were treated with antibiotics and inoculated into L20B (cell-line-expressing poliovirus receptor) and RD cell (human rhabdomyosarcoma) lines, as previously described [4]. Cell cultures exhibiting cytopathic effect (CPE) were harvested and stored at −20 °C until typing. The limit of detection ofthe used method is that it should be able to detect 20 TCID50/sample in accordance with guidelines for environmental surveillance of poliovirus circulation [4].

Viral RNA was extracted from culture supernatant (QIAamp Viral RNA Mini Kit QIAGEN, Santa Clara, CA, USA), and RT-PCR reactions were performed using the primer pairs 292/222 (for partial VP1 amplification) and Y7/Q8 (for poliovirus complete VP1 gene amplification) as previously described [2,32]. Poliovirus isolates should undergo intratypic differentiation, to identify them as wild-type or vaccine-like, as recommended by the guidelines for environmental surveillance of poliovirus circulation [4].

The EV positive amplicons were gel-purified (QIAquick Gel Extraction Kit, QIAGEN, Hilden, Germany) and cycle-sequenced by the Sanger method (ABI PRISM BigDye Terminator v.3.1, Cycle Sequencing Ready Reaction Kit (Applied Biosystems), Bedford, MA, USA). The obtained nucleotide sequences were compared with those available at GenBank.

For phylogenetic reconstruction, representative sequences were selected according to nucleotide similarity (BLAST). Sequences were edited using BioEdit software version 7.2.5 and aligned using MAFFT version 7 [33]. Phylogenetic trees were reconstructed using a maximum likelihood method with the general time-reversible (GTR) + Gamma substitution model in RAxML version 8.2.11 software [34]. The generated tree was visualized and personalized in the FigTree version 1.4.3. Sequences were deposited at the GenBank (NCBI), under the accession numbers: MN701060–61, MN701064–66, MN701068–70, MN745077, MN888071–86.

## 5. Conclusions

Our findings provide valuable information about the EV environmental circulation pattern in Maputo city in the One Health approach. Although no VDPV was detected in the current study, the ES program in Mozambique must address critical issues about surveillance activity and the feasibility of expanding to all geographic regions of the nation considering a recent upsurge in VDPV and wild poliovirus cases in this African nation.

## Figures and Tables

**Table 1:** Enterovirus identified from sewage samples by collection site.

Month	Location	No of Samples Collected by Site	Isolation by RD or L20B (Positive Specimens)	Intratypic Differentiation *	Typing
January	ETAR (2) Marginal Avenue (1)	2	RD	Negative	CVB5, E7 CVB5
February	ETAR (1)	2	RD and L20B	Positive	PV1
March	ETAR (1)	2			E11
	Marginal Avenue (1) November 10th Avenue (1)		RD	Negative	EV-B75 CVB5
April	ETAR (1) November 10th Avenue (1)	2	RD	Negative	E7 E11
May	Marginal Avenue (1) November 10th Avenue (2)	2	RD	Negative	E11 E11 (2)
June	ETAR (1)	2	RD	Negative	CVB3
July	ETAR (2)	2	RD	Negative	CVB3, E6
August	ETAR (1) November 10th Avenue (1)	2	RD	Negative	CVB3 E11
September	ETAR (2) November 10th Avenue (1)	2	RD	Negative	CVB3, E27 CVB3
October	ETAR (1) Marginal Avenue (2)	2	RD	Negative	E11 CVB3 (2)
November	ETAR (1) Marginal Avenue (1)	1	RD	Negative	CVB3 CVB3

* Molecular screening tool able to detect the presence of poliovirus; ETAR = Infulene sewage treatment plant; ( ) in brackets is the number of isolates; CVB5 = coxsackievirus B5; CVB3 = coxsackievirus B3; EV-B75 = enterovirus B75; E7 = echovirus 7; E11 = echovirus 11; E6 = echovirus 6; PV1 = poliovirus 1.

## Data Availability

All data generated or analyzed during this study are included in this published article.
